# Unmet Needs for Rehabilitative Management in Common Health-Related Problems Negatively Impact the Quality of Life of Community-Dwelling Stroke Survivors

**DOI:** 10.3389/fneur.2021.758536

**Published:** 2021-12-23

**Authors:** Kyoung Tae Kim, Won Kee Chang, Yun-Sun Jung, Sungju Jee, Min Kyun Sohn, Sung-Hwa Ko, Yong-Il Shin, Ja-Ho Leigh, Won-Seok Kim, Nam-Jong Paik

**Affiliations:** ^1^Department of Rehabilitation Medicine, Seoul National University Bundang Hospital, Seoul National University College of Medicine, Seongnam, South Korea; ^2^Department of Rehabilitation Medicine, Keimyung University Dongsan Hospital, Keimyung University School of Medicine, Daegu, South Korea; ^3^Department of Rehabilitation Medicine, Chungnam National University College of Medicine, Chugnam National University Hospital, Daejeon, South Korea; ^4^Department of Rehabilitation Medicine, Pusan National University School of Medicine, Pusan National University Yangsan Hospital, Yangsan, South Korea; ^5^Department of Rehabilitation Medicine, Seoul National University Hospital, Seoul, South Korea; ^6^National Traffic Injury Rehabilitation Research Institute, National Traffic Injury Rehabilitation Hospital, Yangpyeong, South Korea

**Keywords:** stroke, unmet need, community-dwelling, rehabilitation, post-stroke checklist, quality of life, transitional care

## Abstract

**Purpose:** Community-dwelling stroke survivors have various unmet needs for rehabilitative management, but there is a lack of in-depth investigations on common health problems after stroke. Moreover, the association between unmet needs and health-related quality of life (HRQoL) has not been thoroughly investigated. This study aimed to investigate the unmet needs for rehabilitative management in common problems after stroke and their associations with HRQoL among community-dwelling stroke survivors.

**Methods:** A face-to-face cross-sectional survey was conducted among community-dwelling stroke survivors visiting outpatient clinics of rehabilitation departments between June and October 2020 in three university-affiliated hospitals. Unmet needs for common problems after stroke were assessed across eight domains based on the post-stroke checklist: spasticity, dysphagia, communication, cognition, ambulation, pain/discomfort, anxiety/depression, and self-care. HRQoL was measured using the EuroQoL-5D three level (EQ-5D). The prevalence of unmet needs for rehabilitative management and their associations with the EQ-5D index were analyzed.

**Results:** Among the 239 participants who responded to the survey, 63% (*n* = 150) were men. The mean age was 63 ± 13 years, and the mean duration of stroke onset was 55.6 months. Overall, 49% reported at least one unmet need, and the most frequently reported unmet needs were anxiety/depression (20.9%), self-care (20.9%), and pain/discomfort (18.0%). The highest proportion of unmet needs was in the anxiety/depression, communication, and cognition domains. Patients with unmet needs for cognition and pain/discomfort showed a significantly lower EQ-5D index, even after adjusting for age, sex, and modified Rankin scale scores. The total number of unmet needs was significantly correlated with a lower EQ-5D index (Pearson's *r* = −0.329, *p* < 0.001) in the multivariate linear regression model.

**Conclusions:** Unmet rehabilitative needs are prevalent among community-dwelling stroke survivors, and the proportion of unmet needs was high among non-physical domains such as anxiety/depression. The number of unmet needs is an independent negative predictor of HRQoL. Systematic approaches to identify unmet needs and provide appropriate rehabilitative management are required in long-term stroke survivors.

## Introduction

Stroke is one of the leading causes of mortality and long-term physical, psychological, and social disabilities (1, 2). Although the incidence of stroke is increasing, its mortality has been decreasing owing to improved acute stroke care. Consequently, the number of stroke survivors living with disabilities who need long-term care is also increasing (2–4) and its socioeconomic burden (5).

Stroke survivors have various long-term health problems, such as reduced physical functioning (6), spasticity (7), memory loss (8), urinary incontinence (9), communication (10), and mood (11), which can lead to increased socioeconomic burden, participation restriction, and worse health-related quality of life (HRQoL) (12). These long-term health problems in stroke survivors are often not properly managed and remain as unmet needs. A national survey of long-term needs of stroke survivors in the UK showed that over half of patients have unmet needs (13). Andrew et al. also demonstrated that 84% of stroke survivors reported having unmet needs at a median 2 years after stroke (14). Accordingly, various studies have investigated a wide range of multidimensional unmet needs after stroke (15–20). However, previous studies did not focus on the unmet need for care in specific health-related problems after stroke.

In this study, we developed a questionnaire based on a post-stroke checklist (PSC) to assess unmet needs for rehabilitation management in common health-related problems after stroke. The PSC is a set of questionnaires developed by the Global Stroke Community Advisory Panel to identify long-term problems in stroke survivors (21). It examines 11 domains of long-term problems, including, secondary prevention, activities of daily living, mobility, spasticity, pain, incontinence, communication, mood, cognition, life after stroke, and relationships with caregivers. It focuses on the areas where intervention can have a large impact on HRQoL and has been recognized as a standardized tool for assessing long-term unmet needs in clinical practice (22).

In addition, although there have been many reports on the prevalence of unmet needs among stroke survivors, only few studies have investigated their association with HRQoL. Im et al. reported that worsening of mobility and communication problems was significantly associated with worse HRQoL in stroke survivors at the 12-month follow-up (23). Andrew et al. (24) also reported that pain and activity limitation were related to long-term unmet needs at 90 and 180 days following stroke. Nonetheless, the prevalence of long-term unmet needs and their impact on HRQoL remains unclear among chronic community-dwelling stroke survivors. Additionally, identifying the prevalence of unmet needs and their relationship with HRQoL using a conveniently administrable questionnaire will help physicians quickly recognize unmet needs and provide proper management in clinical settings.

This study aimed to investigate the unmet needs for rehabilitation services in the domains of common health problems after stroke in community-dwelling stroke survivors and establish the relationship between unmet needs for rehabilitative management and HRQoL. To achieve this goal, we developed a questionnaire based on the PSC for unmet needs, specifically for the rehabilitative management of common health-related problems after stroke.

## Materials and Methods

### Participants and Study Design

This study included 239 community-dwelling post-stroke patients aged over 18 years who visited the outpatient clinics of the Department of Rehabilitation Medicine at Seoul National University Bundang Hospital (Seongnam-si, Gyeonggi-do, Korea), Chungnam National University Hospital (Jung-gu, Daejeon, Korea), and Pusan National University Yangsan Hospital (Yangsan-si, Gyeongsangnam-do, Korea) between June and October 2020. The cross-sectional survey was conducted using structured questionnaires and face-to-face interviews of the patients or their proxies (e.g., parents, spouse, siblings, children, relatives, and other caregivers). The surveyors provided an oral presentation and obtained informed consent from the patient before the interview. If the patients could not participate due to severe cognitive or communication impairments, the oral presentation and written informed consent form were provided to the proxies. When proxies responded to the questionnaires, they were instructed to answer each question on behalf of the participant.

This study was approved by the Institutional Review Board of Seoul National University Bundang Hospital (IRB No. B-1910/572-303). All participants provided written informed consent.

### Measures

#### Unmet Needs for Rehabilitative Management

A survey questionnaire was developed to investigate the unmet needs for rehabilitation management of common post-stroke problems based on the PSC (21). Eight items (i.e., spasticity, dysphagia, communication, cognition, ambulation, pain/discomfort, mood [anxiety/depression], and self-care) were included in the survey. Through the questionnaire, the respondents were asked whether they have a need for rehabilitative management regarding each category. If the participants had a need for such, we asked whether sufficient treatment was received. Unmet needs were defined as a response of not receiving sufficient rehabilitative managements despite having the need for such. Meanwhile, met needs were defined as a participant response of not needing rehabilitative managements or receiving sufficient managements. The total number of unmet needs for each participant was then calculated.

#### Quality of Life

The HRQoL of participants was measured using the EuroQoL-5D three level (EQ-5D-3L). EQ-5D-3L consists of five domains: mobility, self-care, usual activity, pain/discomfort, and anxiety/depression (25). Each domain is reported on a three-point Likert scale: no problem, moderate problem, and severe problem (25). The EQ-5D index was calculated using the Korean valuation set (26, 27).

#### Other Variables

Baseline demographics, including age, sex, marital status (married, widow/separated, single), living alone (yes/no), household income, type of health insurance (national health insurance [employed], and national health insurance [self-employed], medical aid) were obtained. The clinical data of participants, including time since the onset of stroke, type of stroke (ischemic or hemorrhagic), and modified Rankin Scale score (mRS) (0 to 2 / 3 to 6) assessed using a simplified mRS questionnaire (28), were investigated. The degree of disability was also surveyed and categorized as mild (grades 4–7), severe (grade 1–3), or not registered. The post-stroke grade of disability was determined based on the Korean version of the modified Barthel index score (29). We also asked the participants whether they experienced unexpected re-admission within 3 months after home discharge.

### Statistical Analyses

The baseline characteristics are presented as numbers and percentages. The prevalence of needs and unmet needs for rehabilitative management in common problems after stroke and unexpected readmission within 3 months after home discharge were also presented as numbers and percentages. Analysis of covariance adjusting for age, sex, and mRS was performed to compare the EQ-5D index per problem between the met and unmet need groups.

To determine the final model for the EQ-5D index, univariate analyses were initially performed using a single linear regression. The EQ-5D index was set as a dependent variable, while baseline characteristics and the total number of unmet needs were set as independent variables. Then, significant variables in the univariate analyses (*p*-value < 0.05) were entered into a forward stepwise multiple linear regression model, with entry condition of *p*-value < 0.05 and removal condition of *p*-value > 0.10. Age was included as a continuous variable, while all other characteristics were included as categorical variables. All models were tested for collinearity, and a variance inflation factor <10 was considered acceptable. Pearson correlation analysis was performed to investigate the correlation between the number of unmet needs and the EQ-5D index. All analyses were conducted using SPSS v.21.0 (IBM Corp, Armonk, NY, USA), and a two-sided *p*-value of < 0.05 was considered statistically significant.

## Results

### Participant Characteristics

A total of 239 stroke survivors completed the survey. Responses were provided directly by 147 patients (61.5%) and 92 proxies (38.5%). The baseline characteristics are presented in Table 1. The mean age was 63.0 ± 12.9 years, and 62.8% (*n* = 150) were men. Approximately 77.0% (*n* = 184) of the participants were in the chronic phase (i.e., more than 1 year after stroke onset), with a mean stroke onset duration of 55.7 ± 51.1 months. The mean mRS was 2.7 ± 1.7, and nearly half (*n* = 107) belonged to a low-income household (i.e., monthly incomes of ≤ 2 million won).

**Table 1:** Baseline patient characteristics (*n* = 239).

**Characteristics**	***n***	**(%)**
**Age, years**		
<40	11	(4.6)
40–59	79	(33.1)
60–69	70	(29.3)
≥70	79	(33.1)
**Sex**		
Male	150	(62.8)
Female	89	(37.2)
**Time since stroke^a^, years**		
<1	53	(22.4)^a^
1 to <5	78	(32.6)^a^
5 to <10	81	(34.2)^a^
≥10	25	(10.5)^a^
**Type of stroke** ^**b**^		
Ischemic	139	(58.9)^b^
Hemorrhagic	97	(41.1)^b^
**Modified Rankin Scale score** ^**c**^		
0–2	112	(47.1)^c^
3–6	126	(53.9)^c^
**Household income (KRW)/month)** ^**d**^		
<2000	107	(45.5)^d^
2000 to <3000	61	(26.)^d^
3000 to <5000	45	(19.1)^d^
5000 to <7000	12	(5.1)^d^
≥7000	10	(4.3)^d^
**Health insurance** ^**c**^		
National health insurance (employed)	86	(36.1)^c^
National health insurance (self-employed)	125	(52.5)^c^
Medical aid	27	(11.3)^c^
**Marital status** ^**c**^		
Married	158	(66.4)^c^
Widow or separated	51	(21.4)^c^
Single	29	(12.2)^c^
**Living alone** ^**a**^		
Yes	36	(15.2)^a^
No	201	(84.8)^a^
**Disability grade** ^**a**^		
Severe	83	(35.0)^a^
Mild	61	(25.7)^a^
Not registered	93	(39.2)^a^

a *n = 237;*

b *n = 236;*

c *n = 238;*

d *n = 235*.

### Unmet Needs for Rehabilitative Managements

**Figure 1:**
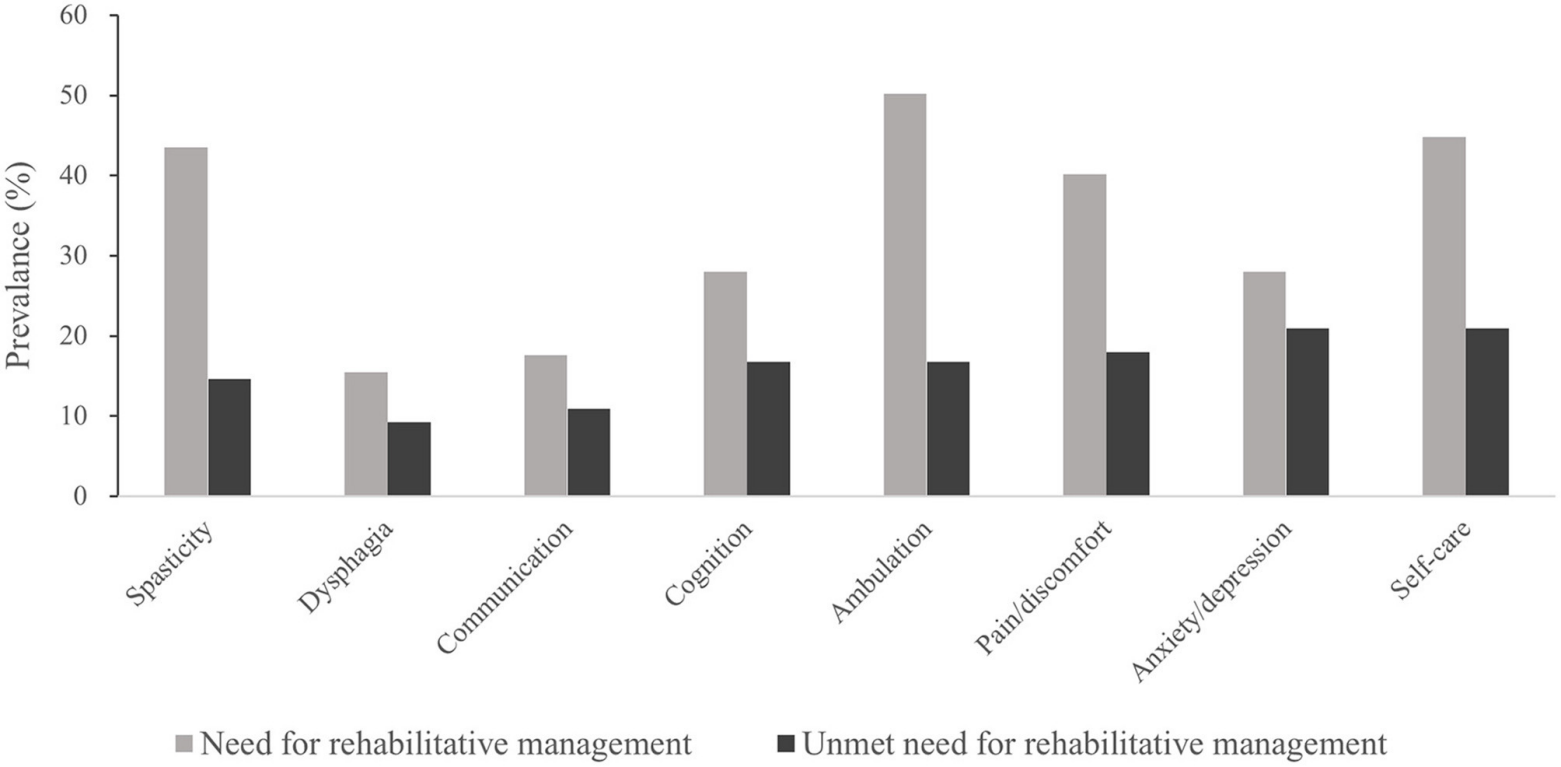
Prevalence of met and unmet needs according to the common problems after stroke.

In total, 118 (49.3%) participants reported at least one unmet need for rehabilitative management, with a mean of 2.6 ± 2.0 participants. The most prevalent need for rehabilitative management was for ambulation (50.2%), followed by self-care (44.8%), spasticity (43.5%), and pain/discomfort (42.1%). The most prevalent unmet needs were for anxiety/depression (20.9%), self-care (20.9%), and pain/discomfort (18.0%) (Figure 1). The proportion of unmet needs was the highest in the anxiety/depression domain (74.6%), followed by communication (61.9%) and cognition (59.7%).

### Unmet Needs for Rehabilitative Managements and HRQoL

**Figure 2:**
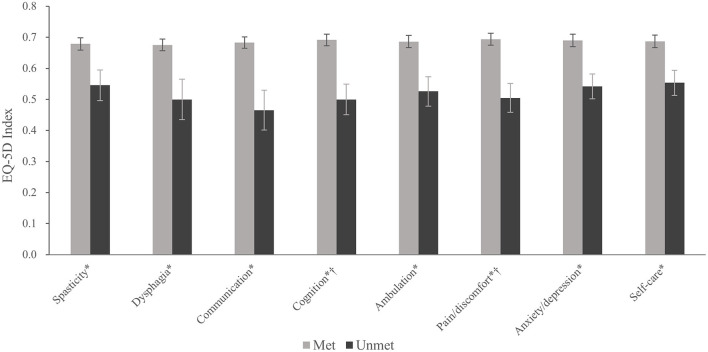
EQ-5D index according to the presence of unmet need for each common problem after stroke. Values are presented as the mean and standard error. *p < 0.05, after adjusting for age and sex. *^†^* *p* < 0.05, after adjusting for age, sex, and modified Rankin Scale.

The unmet need group showed worse HRQoL than the met need group across all domains after adjusting for age and sex (Figure 2). Additionally, after adjusting for mRS, unmet needs for cognition and pain/discomfort were associated with worse HRQoL.

The increased number of unmet needs was associated with a lower EQ-5D index (Pearson's *r* = −0.329, *P* < 0.001). Five variables were fit in our final model (*R*^2^ = 0.602, *F* = 67.73, *P* < 0.001). Among them, mRS, age, and the number of unmet needs showed significant negative correlations with the EQ-5D index, while disability grade and household income showed significant positive correlations with the EQ-5D index (Table 2).

**Table 2:** Final multiple linear regression model for health-related quality of life.

**Variables**	**β ± SE**	**95% CI**	***p*-value**
Modified Rankin Scale score	−0.099, 0.009	−0.117, −0.082	<0.001
Disability grade^a^	0.072, 0.017	0.038, −0.105	<0.001
Household income^b^	0.027, 0.011	0.004, 0.049	0.020
Age	−0.002, 0.001	-0.004, 0.000	0.035
Number of unmet needs	−0.015, 0.007	−0.028, −0.001	0.037

*F = 67.73 (p < 0.001), Adjusted R^2^ for model = 0.602*.

a *Disability grade is defined as an ordinal variable in the following order: severe/mild/not registered*.

b *Household income is defined as an ordinal variable in the following order: ≤ 2000 KRW/2000-3000 KRW/3000-5000 KRW/5000-7000 KRW/≥7000 KRW*.

### Unexpected Readmission Within 3 Months After Home Discharge

In total, 17.6% (*n* = 42) of the participants were readmitted within 3 months after home discharge. Among them, 23.8% (*n* = 10) were re-admitted due to stroke-related problems (e.g., stroke recurrence, decline in activities of daily living, pressure ulcers, and infections); 37.5% (*n* = 15), internal and surgical reasons; and another 37.5% (*n* = 15), other non-medical reasons such as difficulty in home care and transfer.

## Discussion

The association between unmet needs and HRQoL among community-dwelling stroke survivors remains unclear. In this study, 49.3% of the community-dwelling stroke survivors had at least one unmet need for rehabilitative management in common problems after stroke. Stroke survivors who had unmet needs, specifically for cognition and pain/discomfort showed worse HRQoL, even after adjusting for age, sex, and mRS. The total number of unmet needs was a significant predictor of worse HRQoL in the final multiple linear regression model.

The prevalence of unmet needs in our study was consistent with those of previous studies (13, 14); however, it was also significantly lower than those of other studies that reported >70% prevalence of unmet needs (15, 17, 19). A recent review article showed that the prevalence of unmet needs among stroke survivors varies widely from 19.8 to 91.7% (30). We assumed that this heterogeneity could be due to the differences in patient demographics, definition of unmet needs, and scope of the assessment tools used. In this study, we defined “unmet need” as a post-stroke problem that needs rehabilitative management but is not treated sufficiently. Meanwhile, other studies defined “unmet needs” to a wider extent, including no or insufficient help for difficulty or a problem that has not been addressed sufficiently (14, 19). Moreover, our study focused on identifying health-related unmet needs; thus, the unmet needs in other domains, such as leisure, work, and other socioeconomic domains were not included.

The mean age of the participants in our study was 63 years, which is younger than that of previously reported studies by Andrew et al. and Mckevitt et al. (13, 14). This may be related to possible selection bias. However, similar results have been reported in tertiary hospital outpatient clinics (31). Concerning the relationship between age and unmet needs, Kersten et al. (32) reported no difference in the number of unmet needs among age groups. Andrew et al. (14) have reported that age is negatively associated with unmet needs in the living, support, and financial categories, but not regarding health. Our study investigated health-related unmet needs and did not show a significant association between unmet needs and age.

Among the various health-related needs of stroke survivors, ambulation, self-care, and spasticity were the most frequently identified rehabilitative needs. These physical problems are associated with motor impairment, which is a common sequela of stroke (7, 33, 34). Moreover, these problems have been reported to be prominent in community-dwelling environments than in hospitals (23), resulting in a high prevalence of rehabilitative management needs among community-dwelling stroke survivors. In this study, the most frequently reported unmet rehabilitative need was for self-care, followed by anxiety/depression and pain/discomfort. Among participants who needed rehabilitative management, the highest proportion of unmet needs was for anxiety/depression (74.6%), followed by communication (61.9%) and cognition (59.7%). These results imply that non-physical needs were less likely to be met than physical needs, despite physical needs being more common. For example, in the domains of spasticity and ambulation, the rate of met needs was high, while the rate of unmet needs was low. However, contradicting results were obtained in the domains of anxiety/depression, communication, and cognition.

Under-recognition of non-physical problems might be one explanation for the high proportion of unmet rehabilitative needs. More than 50% of patients experience emotional disturbances after stroke (35), and these are associated with worsened quality of life and increased burden on caregivers (36, 37). Despite its high prevalence, anxiety/depression problems have not been adequately addressed (14). A national audit of inpatient rehabilitation in Australia reported that approximately only half of stroke patients received an assessment for mood (38). For communication problems, up to one-third of stroke survivors experience communication difficulties, such as aphasia (39). Communication problems in stroke survivors are highly related to their psychosocial well-being; (40) thus, understanding the full impact of communication disorders using a traditional approach based on specific impairments may be insufficient (41). Lastly, cognitive problems negatively impact self-esteem, confidence, and functional recovery, consequently increasing the long-term burden of stroke (42). Despite its importance, the underlying nature of cognitive problems is less understood than that of physical problems, including among clinicians and caregivers (43). Hence, these non-physical problems are often not recognized sufficiently and require more attention from clinicians.

The mean EQ-5D index was significantly lower in patients with unmet needs after adjusting for age and sex in all domains. However, only cognition and pain/discomfort showed a significantly lower EQ-5D index after additionally adjusting for mRS. Cognitive impairment negatively impacts the quality of life of stroke survivors (44–46). Pain is also known to be associated with HRQoL. Choi-Kwon et al. reported that musculoskeletal and central pain were closely associated with HRQoL at 1 year post-stroke, and the presence of central post-stroke pain was an important explanatory factor for overall QoL 3 years after stroke (47, 48). Notably, we found that these two domains were related to the EQ-5D index after adjusting for mRS, implying that unmet needs for cognition and pain/discomfort are associated with worse HRQoL independent of the respondents' overall functional status.

The number of unmet needs showed a significant negative correlation with the mean EQ-5D index. The Australian Stroke Clinical Registry reported a similar trend in which a negative exponential relationship was observed between the EQ-5D VAS scores and the number of unmet needs between 90 and 180 days after stroke (24). Our study results confirmed that this negative correlation is also applicable among long-term community-dwelling stroke survivors. Multivariate linear regression analysis showed that the number of unmet needs along with mRS, household income, degree of disability registration, and marital status were significant factors correlated with HRQoL. This result demonstrates the importance of recognizing the unmet needs of community-dwelling stroke survivors and providing proper intervention, as the number of unmet needs is negatively correlated with HRQoL.

Among the community-dwelling stroke survivors, 17.5% had unexpected re-admission within 3 months after home discharge; in total, 35 and 30% of these readmissions were due to internal or surgical medical causes and non-medical causes, respectively. Our study's unexpected re-admission rate was higher than those reported in the previous studies. Two reasons may contribute to this finding. First, in the study by the Kilkenny et al. (49) and Lin H-J et al. (50), readmission due to non-medical causes was excluded. In our case, non-medical cause such as difficulty of patient care accounted for 37.5% of total unexpected re-admissions. After excluding readmission due to non-medical causes, the unexpected re-admission rate is dropped to 11% (27 out of 239 participants). Additionally, considering the survey was performed in the outpatient setting of tertiary hospitals, our study participants may have greater disability severity than those of previous studies. Kilkenny et al. (49) have identified limited access to information, health, and community services as a risk factor for re-admission after home discharge in stroke patients. Moreover, caregiver burden is an important factor for re-admission, especially for those due to non-medical causes. Many caregivers of community-dwelling stroke survivors have increased burden, which negatively affects the patient's quality of life, leading to re-admission (51, 52). Providing adequate social support to lower the burden of caregivers and increasing accessibility to information and community services may be helpful in reducing unexpected readmissions.

Selection bias may have occurred in our study, as it was conducted at the outpatient clinics of tertiary hospitals. Thus, the survivors who required rehabilitative management but could not visit the clinic due to accessibility issues, including those with a severe disability who do not have a caregiver to bring the patient to the clinic, poor economic status, and long-term institutionalization, were likely to be excluded. However, in our study, the percentage of participants with mRS of 3–5 was 53.9%, which is higher than the results of a 3-month mRS from a multicenter study in Korea (53), signifying that our sample may represent the patients who could visit the rehabilitation outpatient clinic, regardless of their disability severity.

Moreover, the possibility of bias due to responder-type may also exist. Several studies have reported that proxy-responders report more disabilities and worse HRQoL than patients (54, 55). We have investigated whether there is a difference in the prevalence of unmet needs according to the responder type (participant vs. proxy-responder) and found none in terms of communication and cognition. The correlation between EQ-5D-3L and mRS, EQ-5D-3L with the number of unmet needs was also similar between the participant and surrogate responder groups, implying that the assessment of proxy-responders was comparable to that of the participants. Our study has a few other limitations. First, our survey did not cover the comprehensive scope of unmet needs, including social activities, finances, housing, community services, and employment. However, the in-depth investigation of unmet needs in a specific domain of rehabilitation management overcame these limitations and provided additional information. Second, the definition of an unmet need in our study was based on the participant's self-reported questionnaire and did not include additional information regarding the actual dosage or quality of rehabilitation management received by the participants. Therefore, although we identified the patient's unmet needs subjectively, we were unable to differentiate between unmet and unresolved needs based on the questionnaire. Third, our study did not investigate the comorbidities of the participants. It has been reported that concurrent comorbidities are negatively associated with HRQoL (56). Our study examined the relationship between the number of unmet needs and HRQoL along with functional impairment, disability, age, and income, which have been reported as independent determinants of long-term HRQoL in stroke survivors (12). Finally, a causal relationship between unmet needs and HRQoL could not be established because of the limitations of the cross-sectional study design.

Nonetheless, the strength of our study is that we implemented an easily administrable self-reported questionnaire to identify long-term unmet needs in various categories, which may be improved with proper intervention. The results demonstrate a high prevalence of unmet needs in non-physical categories of long-term stroke survivors. Physicians can easily recognize these unmet needs using a questionnaire similar to that proposed in this study. Considering non-physical unmet needs are often underrecognized in clinical settings, our study emphasizes the demand to identify these unmet needs and plan for appropriative rehabilitation management in out-patient clinics. In conclusion, among community-dwelling stroke survivors, there is a high proportion of unmet needs in non-physical domains, such as anxiety/depression, communication, and cognition. Further, the number of unmet needs is an independent predictor of HRQoL. Systematic approaches to identify unmet needs and provide appropriate rehabilitative management are required in long-term stroke survivors.

## Data Availability Statement

The original contributions presented in the study are included in the article/supplementary material, further inquiries can be directed to the corresponding author/s.

## Ethics Statement

The studies involving human participants were reviewed and approved by Institutional Review Board of Seoul National University Bundang Hospital. The patients/participants provided their written informed consent to participate in this study.

## Author Contributions

KK, WC, SJ, MS, S-HK, Y-IS, J-HL, W-SK, and N-JP: conceptualization. KK, WC, Y-SJ, J-HL, MS, Y-IS, W-SK, and N-JP: methodology. KK, WC, Y-SJ, W-SK, and N-JP: validation/formal analysis. KK, WC, Y-SJ, SJ, MS, S-HK, Y-IS, W-SK, and N-JP: investigation. KK and WC: writing–original draft. KK, WC, Y-SJ, SJ, MS, S-HK, Y-IS, J-HL, W-SK, and N-JP: writing–review and editing. MS, Y-IS, W-SK, and N-JP: project administration. N-JP: funding acquisition. All authors contributed to the article and approved the submitted version.

## Funding

This work was supported by the Research Program funded by the Korea National Institute of Health (2020ER630601).

## Conflict of Interest

The authors declare that the research was conducted in the absence of any commercial or financial relationships that could be construed as a potential conflict of interest.

## Publisher's Note

All claims expressed in this article are solely those of the authors and do not necessarily represent those of their affiliated organizations, or those of the publisher, the editors and the reviewers. Any product that may be evaluated in this article, or claim that may be made by its manufacturer, is not guaranteed or endorsed by the publisher.

## References

[1] Murray J Young J Forster A. Measuring outcomes in the longer term after a stroke. Clin Rehabil. (2009) 23:918–21. 10.1177/0269215509341525 19779006

[2] Feigin VL Norrving B Mensah GA. Global burden of stroke. Circ Res. (2017) 120:439–48. 10.1161/CIRCRESAHA.116.308413 28154096

[3] Katan M Luft A. Global burden of stroke. Semin Neurol. (2018) 38:208–11. 10.1055/s-0038-1649503 29791947

[4] Kim JS. Stroke in Asia: a global disaster. Int J Stroke. (2014) 9:856–7. 10.1111/ijs.12317 25231579

[5] Kim JS. Stroke becomes the 3rd important cause of death in Korea; is it a time to toast? J Stroke. (2014) 16:55. 10.5853/jos.2014.16.2.55 24949308 PMC4060274

[6] Hartman-Maeir A Soroker N Ring H Avni N Katz N. Activities, participation and satisfaction one-year post stroke. Disabil Rehabil. (2007) 29:559–66. 10.1080/09638280600924996 17453976

[7] Urban PP Wolf T Uebele M Marx JJ Vogt T Stoeter P. Occurence and clinical predictors of spasticity after ischemic stroke. Stroke. (2010) 41:2016–20. 10.1161/STROKEAHA.110.581991 20705930

[8] Gamaldo A Moghekar A Kilada S Resnick SM Zonderman AB O'Brien R. Effect of a clinical stroke on the risk of dementia in a prospective cohort. Neurology. (2006) 67:1363–9. 10.1212/01.wnl.0000240285.89067.3f 17060561

[9] Wissel J Olver J Sunnerhagen KS. Navigating the poststroke continuum of care. J Stroke Cerebrovasc Dis. (2013) 22:1–8. 10.1016/j.jstrokecerebrovasdis.2011.05.021 21733720

[10] Bertheir ML. Poststroke aphasia: epidemiology, pathophysiology and treatment. Drugs Aging. (2005) 22:163–82. 10.2165/00002512-200522020-00006 15733022

[11] Hackett ML Anderson CS House AO. Management of depression after stroke: a systematic review of pharmacological therapies. Stroke. (2005) 36:1092–7. 10.1161/01.STR.0000162391.27991.9d 15802637

[12] Sturm JW Donnan GA Dewey HM Macdonell RAL Gilligan AK Srikanth V. Quality of life after stroke: the north east melbourne stroke incidence study (NEMESIS). Stroke. (2004) 35:2340–5. 10.1161/01.STR.0000141977.18520.3b 15331799

[13] McKevitt C Fudge N Redfern J Sheldenkar A Crichton S Rudd AR. Self-reported long-term needs after stroke. Stroke. (2011) 42:1398–403. 10.1161/STROKEAHA.110.598839 21441153

[14] Andrew NE Kilkenny M Naylor R Purvis T Lalor E Moloczij N. Understanding long-term unmet needs in Australian survivors of stroke. Int J Stroke. (2014) 9:106–12. 10.1111/ijs.12325 25042019

[15] Forster A. Validation of the longer-term unmet needs after stroke (luns) monitoring tool: a multicentre study. Clin Rehabil. (2013) 27:1020–8. 10.1177/0269215513487082 23787941

[16] Groeneveld IF Arwert HJ Goossens PH Vliet Vlieland TPM. The longer-term unmet needs after stroke questionnaire: cross-cultural adaptation, reliability, and concurrent validity in a dutch population. J Stroke Cerebrovasc Dis. (2018) 27:267–75. 10.1016/j.jstrokecerebrovasdis.2017.08.043 28967592

[17] Rothwell K Boaden R Bamford D Tyrrell PJ. Feasibility of assessing the needs of stroke patients after six months using the GM-SAT. Clin Rehabil. (2013) 27:264–71. 10.1177/0269215512457403 22952306 PMC3652600

[18] van de Port IGL van den Bos GAM Voorendt M Kwakkel G Lindeman E. Identification of risk factors related to perceived unmet demands in patients with chronic stroke. Disabil Rehabil. (2007) 29:1841–6. 10.1080/09638280601129157 17852229

[19] Olaiya MT Cadilhac DA Kim J Nelson MR Srikanth VK Andrew NE. Long-term unmet needs and associated factors in stroke or TIA survivors. Neurology. (2017) 89:68–75. 10.1212/WNL.0000000000004063 28566545

[20] Ullberg T Zia E Petersson J Norrving B. Perceived unmet rehabilitation needs 1 year after stroke: an observational study from the swedish stroke register. Stroke. (2016) 47:539–41. 10.1161/STROKEAHA.115.011670 26732564

[21] Philp I Brainin M Walker MF Ward AB Gillard P Shields AL. Development of a poststroke checklist to standardize follow-up care for stroke survivors. J Stroke Cerebrovasc Dis. (2013) 22:e173–80. 10.1016/j.jstrokecerebrovasdis.2012.10.016 23265778

[22] Ward AB Chen C Norrving B Gillard P Walker MF Blackburn S. Evaluation of the post stroke checklist: a pilot study in the United Kingdom and Singapore. Int J Stroke. (2014) 9:76–84. 10.1111/ijs.12291 25088427

[23] Im HW Kim WS Kim SY Paik NJ. Prevalence of worsening problems using post-stroke checklist and associations with quality of life in patients with stroke. J Stroke Cerebrovasc Dis. (2020) 29:1–8. 10.1016/j.jstrokecerebrovasdis.2020.105406 33254377

[24] Andrew NE Kilkenny MF Lannin NA Cadilhac DA. Is health-related quality of life between 90 and 180 days following stroke associated with long-term unmet needs? Qual Life Res. (2016) 25:2053–62. 10.1007/s11136-016-1234-5 26847339

[25] Group TE. EuroQol - a new facility for the measurement of health-related quality of life. Health Policy. (1990) 16:199–208. 10.1016/0168-8510(90)90421-9 10109801

[26] Jo MW Yun SC Lee S-I. Estimating quality weights for EQ-5D health states with the time trade-off method in South Korea. Value Health. (2008) 11:1186–9. 10.1111/j.1524-4733.2008.00348.x 18489498

[27] Kim MH Cho YS Uhm WS Kim S Bae SC. Cross-cultural adaptation and validation of the Korean version of the EQ-5D in patients with rheumatic diseases. Qual Life Res. (2005) 14:1401–6. 10.1007/s11136-004-5681-z 16047514

[28] Bruno A Akinwuntan AE Lin C Close B Davis K Baute V. Simplified modified rankin scale questionnaire: Reproducibility over the telephone and validation with quality of life. Stroke. (2011) 42:2276–9. 10.1161/STROKEAHA.111.613273 21680905

[29] Criteria For Evaluation Of Degree Of Disability Ministry Of Health And Welfare (2017). Available online at: http://www.law.go.kr/LSW/admRulInfoP.do?admRulSeq=2100000083371. (2019)2019 (accessed October 6, 2019).

[30] Chen T Zhang B Deng Y Fan JC Zhang L Song F. Long-Term unmet needs after stroke: systematic review of evidence from survey studies. BMJ Open. (2019) 9:e028137 10.1136/bmjopen-2018-028137 31110106 PMC6530326

[31] H KK Y YS Tock Seng Hospital T Tan Tock Seng J Keng He Ang Mo Kio K. Health-related quality of life among chronic stroke survivors attending a rehabilitation clinic. Singapore Med J. (2006) 47:213–8. 16518556

[32] Kersten P Low JTS Ashburn A George SL McLellan DL. The unmet needs of young people who have had a stroke: Results of a national UK survey. Disabil Rehabil. (2002) 24:860–6. 10.1080/09638280210142167 12450462

[33] Robinson CA Shumway-Cook A Matsuda PN Ciol MA. Understanding physical factors associated with participation in community ambulation following stroke. Disabil Rehabil. (2011) 33:1033–42. 10.3109/09638288.2010.520803 20923316

[34] Fujita T Iokawa K Sone T Yamane K Yamamoto Y Ohira Y. Effects of the Interaction among Motor Functions on Self-care in Individuals with Stroke. J Stroke Cerebrovasc Dis. (2019) 28:104387. 10.1016/j.jstrokecerebrovasdis.2019.104387 31542365

[35] Ayerbe L Ayis S Crichton S Wolfe CDA Rudd AG. The natural history of depression up to 15 years after stroke: the South London stroke register. Stroke. (2013) 44:1105–10. 10.1161/STROKEAHA.111.679340 23404719

[36] Kim JS Choi-Kwon S Kwon SU Lee HJ Park K-A Seo YS. Factors affecting the quality of life after ischemic stroke: young versus old patients. J Clin Neurol. (2005) 1:59. 10.3988/jcn.2005.1.1.59 20396472 PMC2854931

[37] Choi-Kwon S Kim HS Kwon SU Kim JS. Factors affecting the burden on caregivers of stroke survivors in South Korea. Arch Phys Med Rehabil. (2005) 86:1043–8. 10.1016/j.apmr.2004.09.013 15895355

[38] National Stroke Foundation National Stroke Audit - Rehabilitation Services Report. Melbourne. (2012). Available online at: https://informme.org.au/-/media/D6C49F8C3AB24F81AE3AB341C888CA61.ashx?la=en

[39] Engelter ST Gostynski M Papa S Frei M Born C Ajdacic-Gross V. Epidemiology of aphasia attributable to first ischemic stroke: incidence, severity, fluency, etiology, and thrombolysis. Stroke. (2006) 37:1379–84. 10.1161/01.STR.0000221815.64093.8c 16690899

[40] Worrall L Brown K Cruice M Davidson B Hersh D Howe T. The evidence for a life-coaching approach to aphasia. Aphasiology. (2010) 24:497–514. 10.1080/02687030802698152

[41] Wray F Clarke D. Longer-term needs of stroke survivors with communication difficulties living in the community: a systematic review and thematic synthesis of qualitative studies. BMJ Open. (2017) 7:1–18. 10.1136/bmjopen-2017-017944 28988185 PMC5640038

[42] Barker-Collo S Feigin VL Parag V Lawes CMM Senior H. Auckland stroke outcomes study: part 2: cognition and functional outcomes 5 years poststroke. Neurology. (2010) 75:1608–16. 10.1212/WNL.0b013e3181fb44c8 21041784

[43] Patchick EL Horne M Woodward-Nutt K Vail A Bowen A. Development of a patient-centred, patient-reported outcome measure (PROM) for post-stroke cognitive rehabilitation: qualitative interviews with stroke survivors to inform design and content. Health Expect. (2015) 18:3213–24. 10.1111/hex.12311 25483800 PMC5810690

[44] Cumming TB Brodtmann A Darby D Bernhardt J. The importance of cognition to quality of life after stroke. J Psychosom Res. (2014) 77:374–9. 10.1016/j.jpsychores.2014.08.009 25217449

[45] Caro CC Mendes PVB Costa JD Nock LJ da Cruz DMC. Independence and cognition post-stroke and its relationship to burden and quality of life of family caregivers. Top Stroke Rehabil. (2017) 24:194–9. 10.1080/10749357.2016.1234224 27646977

[46] Pai HC Tsai YC. The effect of cognitive appraisal on quality of life of providers of home care for patients with stroke. J Neurosc Nurs. (2016) 48:E2–11. 10.1097/JNN.0000000000000175 26720323

[47] Choi-Kwon S Choi JM Kwon SU Kang D-W Kim JS. Factors that affect the quality of life at 3 years post-stroke. J Clin Neurol. (2006) 2:34. 10.3988/jcn.2006.2.1.34 20396483 PMC2854941

[48] Choi-Kwon S Choi SH Suh M Choi S Cho KH Nah HW. Musculoskeletal and central pain at 1 year post-stroke: associated factors and impact on quality of life. Acta Neurol Scand. (2017) 135:419–25. 10.1111/ane.12617 27265610

[49] Kilkenny MF Dalli LL Kim J Sundararajan V Andrew NE Dewey HM. Factors associated with 90-day readmission after stroke or transient ischemic attack: linked data from the Australian stroke clinical registry. Stroke. (2020) 51:571–8. 10.1161/STROKEAHA.119.026133 31822248

[50] Lin H-J Chang W-L Tseng M-C. Readmission after stroke in a hospital-based registry Risk, etiologies, and risk factors. Neurology. (2011) 76:438–43. 10.1212/WNL.0b013e31820a0cd8 21209374

[51] Pont W Groeneveld I Arwert H Meesters J Mishre RR Vliet Vlieland T. Caregiver burden after stroke: changes over time? Disabil Rehabil. (2020) 42:360–7. 10.1080/09638288.2018.1499047 30235954

[52] Caro CC Costa JD da Cruz DMC. Burden and quality of life of family caregivers of stroke patients. Occup Ther Health Care. (2018) 32:154–71. 10.1080/07380577.2018.1449046 29578827

[53] Kim SE Lee H Kim JY Lee KJ Kang J Kim BJ. Three-month modified Rankin Scale as a determinant of 5-year cumulative costs after ischemic stroke: an analysis of 11,136 patients in Korea. Neurology. (2020) 94:e978–91. 10.1212/WNL.0000000000009034 32029544

[54] Williams LS Bakas T Brizendine E Plue L Tu W Hendrie H. How valid are family proxy assessments of stroke patients' health-related quality of life? Stroke. (2006) 37:2081–5. 10.1161/01.STR.0000230583.10311.9f 16809575

[55] Carod-Artal FJ Coral LF Trizotto DS Moreira CM. Self-and proxy-report agreement on the stroke impact scale. Stroke. (2009) 40:3308–14. 10.1161/STROKEAHA.109.558031 19661469

[56] Nichols-Larsen DS Clark PC Zeringue A Greenspan A Blanton S. Factors influencing stroke survivors' quality of life during subacute recovery. Stroke. (2005) 36:1480–4. 10.1161/01.STR.0000170706.13595.4f 15947263

